# Time of day dependent changes in embryonic heart rate are detectable after maturation of rhythmic circadian gene expression in the eye, but before the heart in *Xenopus laevis* tadpoles cultured in LD

**DOI:** 10.17912/micropub.biology.001277

**Published:** 2024-09-03

**Authors:** Kristen Curran, Faith Kemper, Morgan Hadley

**Affiliations:** 1 Biology, University of Wisconsin–Whitewater, Whitewater, Wisconsin, United States; 2 Wasseen, Inc. Milwaukee, Wisconsin, United States; 3 University of Kansas Medical Center, Kansas City, Kansas, United States

## Abstract

We systematically characterized onset of expression of circadian genes in the embryonic eye and heart of *Xenopus* *laevis* tadpoles. We found that *period1 (per1)* and *nr1d1* ( *rev-erbα)* were the first circadian genes to display significant 24-hour rhythms in the developing eye and heart in a 12-hour light-dark cycle (LD). Rhythmic expression of both oscillator and output genes were present in the eye by 2.75 days post fertilization (dpf), but not in 15 dpf hearts. Surprisingly, rhythmic oscillation of heart rate occurred after 3.2 dpf suggesting that heart rate may be controlled directly by light or indirectly by the pineal in LD.

**Figure 1. nr1d1 and period1 (per1) were the first genes to display a significant rhythmic expression in the embryonic eye and heart and rhythmic changes in heart rate were detected earlier than expected.**
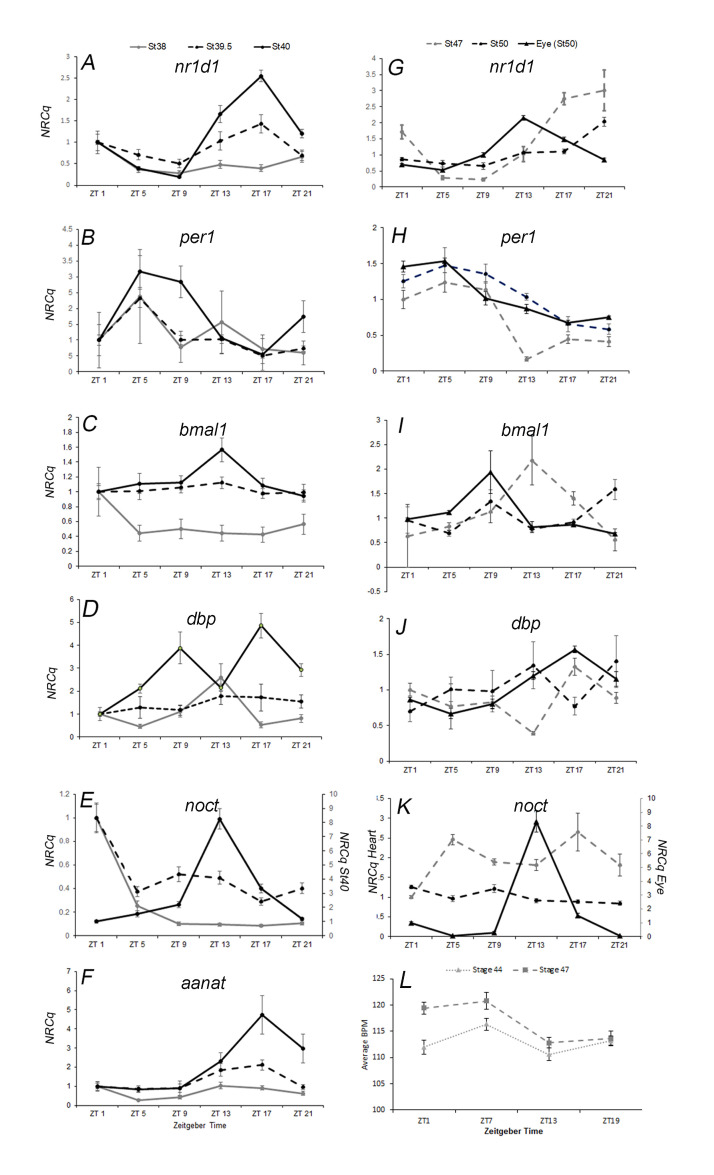
In panels A-F, all circadian genes analyzed in the eye had a significant 24-hour rhythm by stage 40 (2.75 dpf) except *dbp* . Ten eyes from 10 individuals in each sample were dissected in duplicate with representative plots shown here. Four central oscillator genes (A-D) and two clock-controlled genes (E-F) were analyzed. The y-axis indicates normalized relative Cq value (NRCq) and the x-axis indicates the time of day samples were taken during a 12-hour light-dark cycle (Zeitgeber time, ZT). *nr1d1* (A) displayed a significant peak of expression at around ZT17.5 at stage 39.5 (p=0.035, r ^2^ =0.52) and 40 (p=1x10 ^-6^ , r ^2^ =0.99). *per1* (B) displayed a significant peak of expression at approximately ZT6 at stage 38 (p=0.003, r ^2^ =0.92), stage 39.5 (p=0.009, r ^2^ =0.65), and stage 40 (0.00045, r ^2^ =0.93). *bmal1* (C) attained significant rhythmic expression with a peak at ZT12 by stage 40 (p=0.002; r ^2^ =0.89). *dbp* (D) displayed a significant 24-hour rhythm at stage 38 only (p=0.02, r ^2^ =0.67). *noct* (E) and *aanat* (F) displayed a significant 24-hour rhythm only at stage 40. *noct* expression peaked at ZT13 (p=0.0008, r ^2^ =0.91) and *aanat* at ZT17.6 (p=0.02; r ^2^ =0.58). In panels G-K, stage 50 eyes are represented by a solid line, stage 47 hearts by a grey line, and stage 50 hearts by a dashed black line. Stage 50 embryonic eyes were used as a positive control and all genes analyzed displayed a significant circadian rhythm in the eye (p<0.02; solid line). In panels G-K, embryonic hearts had significant rhythmic expression for *nr1d1* (G) and *per1* (H) at stage 47 (5.5 dpf) and stage 50 (15 dpf). *nr1d1* displayed a peak of expression around ZT20 at stage 47 (G; p=0.007, r ^2^ =0.66) and ZT21 at Stage 50 (p=0.0002, r ^2^ =0.67). *per1* displayed a significant peak of expression around ZT 4.75 at stage 47 (H; p=0.0003, r ^2^ =0.83) and at ZT6 at stage 50 (p=0.01, r ^2^ =0.43). All other genes had no significant 24-hour rhythm (I-K; p>0.05). Panel L shows time of day dependent changes in heart rate in tadpoles at two separate developmental timepoints, stage 44 (grey triangle, 3.8 dpf) and stage 47 (grey squares, 5.5 dpf). Heart rate was statistically significant at both stage 44 (peak at ZT 5.6; p=0.004, r ^2^ =0.138, N=23) and stage 47 (peak at ZT 4.73; p=7.1x10 ^-6^ , r ^2^ =0.265, N=22). Error bars represent standard error of the mean from three qPCR replicates. Time of day dependent rhythms were evaluated using Circwave v1.4.

## Description

In most adult cells, the circadian clock coordinates inputs (light, temperature, metabolites) with outputs over a 24-hour period resulting in a circadian rhythm. This coordination is considered evolutionarily adaptive (reviewed by Vaze and Sharma 2013). The molecular oscillator maintains approximately a 24-hour period through a coordinated transcriptional-translational feedback loop. In vertebrates, Bmal1 and Clock dimerize and act as transcription activators for central oscillator genes as well as clock-controlled genes (CCGs). The timing of 24 hours requires that these products feedback on Bmal1/Clock directly (Per1/2, Cry1/2, Dec1 */2* ) or indirectly (Dbp and Nr1d1; reviewed by Patke, et al., 2020). Central oscillator outputs include the CCGs, *nocturnin (noct)* and *aanat,* physiology (melatonin secretion), and behavior (Green and Besharse 1996a; 1996b; Besharse and Iuvone 1983; Koronowski and Sassone-Corsi 2021) . Although, central oscillator components are expressed in most cell types, CCG expression can be cell type specific (Storch et al., 2002) . A hallmark of circadian rhythm is that oscillations can be maintained in constant conditions such as constant darkness (DD, reviewed by Panda et al., 2016; Patke et al., 2020).

We are interested in determining when rhythmic expression of both central oscillator and CCGs are detectable over a 24-hour period in embryonic organs of *Xenopus laevis* (mature molecular circadian clock). In mammals, light input from the eyes informs the suprachiasmatic nucleus (SCN) of the hypothalamus of the time of day. The SCN then synchronizes oscillators in peripheral organs, such as the heart (reviewed by Koronowski and Sassone-Corsi 2021). Zebrafish embryonic organs are light sensitive and thus can reset to light cues independent of the SCN during development (Whitmore et al., 2000; Carr et al., 2006; Weger et al., 2011) . It is not known whether amphibian embryonic organs are light sensitive or not.

Gene expression and melatonin synthesis are used to monitor onset of circadian rhythm in isolated embryonic organs. *nr1d1 (rev-erbα)* and *period1 (per1)* are the first genes to display rhythmic expression in mammalian embryonic pineal gland and suprachiasmatic nucleus (SCN; Sládek et al. 2004; Kováciková et al., 2006; Sládek et al., 2007). In zebrafish the pineal gland displays rhythmic expression of *aanat2* (1 dpf) before the embryonic eye (2.25dpf, Vuilleumier et al., 2006; Ben-Moshe et al., 2014). *aanat1 and 2* encode enzymes involved in melatonin synthesis (reviewed in Zhao et al., 2019). Similarly, isolated *Xenopus laevis* pineal glands exhibit rhythmic changes in melatonin secretion (CCG) in constant darkness (DD) by stage 26 (1.3 days post fertilization, dpf) before isolated eyes (stage 41, 3.2 dpf; Green et al., 1999). We detected a significant rhythm of *bmal1* transcription in isolated eyes of stage 40 *Xenopus laevis* tadpoles cultured in a 12-hour light-dark cycle (LD, 2.75 dpf; Curran et al., 2008).

Less is known about maturation of circadian rhythm in the embryonic heart. Rhythmic central oscillator gene expression is first detectable in mouse and chick hearts at embryonic day 18 (Zeman et al., 2009; Ko et al., 2010; Umemura et al., 2017) . Changes in the rate of heart beat are circadian in three-day old zebrafish maintained in DD (Fong et al., 2021) . We hypothesize that the maturation of a mature molecular circadian clock may indicate an adaptive requirement for an embryonic organ to synchronize with environmental cues (Vaze and Sharma, 2013) .

We systematically performed a developmental time course in LD to assess the onset of rhythmic expression of circadian genes in the embryonic eye and heart of *Xenopus laevis* using qPCR. We found that *per1* and *nr1d1* were the first genes to display a time of day dependent oscillation in developing tadpole eyes and heart when cultured in a 12-hour light-dark cycle (Zeitgeber Time, ZT). ZT0 is dawn and ZT12 is dusk. In the developing eye, *per1* rhythm matured first (stage 38; ~2.25 dpf; p=0.003, r ^2^ =0.92) followed by *nr1d1* (stage 39.5; ~­2.54 dpf; p=0.035, r ^2^ =0.52). *bmal1* was not significantly rhythmic in the eye until stage 40 (2.75dpf ; Figure 1 ; Curran et al., 2008; p=0.002, r ^2^ =0.89). Output genes *noct* and *aanat* also became significantly rhythmic only after stage 40 ( Figure 1 ; p( *noc* )=0.0008, r ^2^ =0.91; p( *aanat* )=0.02, r ^2^ =0.58). One anomaly we observed was the expression of *dbp* , which displayed a significant rhythm at stage 38 (p=0.02, r ^2^ =0.67), but not at stage 39.5 and 40 (p>0.05). Rhythmic expression of *dbp* was significant in stage 50 eyes (15dpf; Figure 1J ; p<0.001, r ^2^ =0.72).

Onset of rhythmic gene expression occurs much later in the hearts of tadpoles cultured in LD. Although earlier stages of development were assessed, we first detected a significant rhythm for *per1* (p=0.0003, r ^2^ =0.83) and *nr1d1* (p=0.0007, r ^2^ = 0.66) in stage 47 embryonic hearts (5.5 dpf). Surprisingly, even after 9.5 more days of development, we again only detected a significant rhythm for *per1* (p=0.01, r ^2^ =0.43) and *nr1d1* (p=0.0002, r ^2^ =0.67) in embryonic hearts (stage 50) *.* Eyes were used as a positive control for these experiments. In stage 50 (15dpf) embryonic eyes, we observed significant circadian rhythms in *nr1d1* (p=0.002, r ^2^ =0.4), *per1* (p=0.006, r ^2^ =0.68) *, bmal1* (p=0.016, r ^2^ =0.42), *dbp (* p<0.001, r ^2^ =0.72) *, noct* (p=0.003, r ^2^ =0.72), and *aanat* (p=0.001, r ^2^ =0.58; Figure 1 ).

Interestingly, we observed significant changes in tadpole heart rate in stages 40-47 tadpoles cultured in LD. In our initial experiments, we measured tadpole heart rate one hour after dawn (ZT1) and one hour after dusk (ZT13) in a 12-hour light dark cycle (N=20-23 tadpoles per time point) at specific stages of development. We observed a significant difference in heart rate between ZT1 and ZT13 in stage 41 (3.17 dpf) and 43 (3.625 dpf) tadpoles (two experiments; two-tailed TTEST, p<0.05). Two more experiments were performed using four time points; ZT1, 7, 13, and 19 to better assess rhythmic changes in heart rate. The change in heart rate between specific times of the day was small (1-2 bpm) but significant at stages 41, 43, 44 (3.8 dpf), and 47 (5.5 dpf) in both experiments (N=22-25 tadpoles per timepoint; p<0.05, r ^2^ =0.13-0.17 for younger hearts and r ^2^ =0.26-0.33 in older hearts). Figure 1L is representative of the time of day dependent changes in heart rate we observed at stage 44 (p=0.004, r ^2^ =0.137; N=24) and stage 47 (p=7.1x10 ^-6^ , r ^2^ =0.265; N=25).

Our findings confirm that rhythmic expression of circadian genes matures gradually and at different developmental times in the eye and heart of *Xenopus laevis* in LD. Circadian genes are expressed during early development (Curran et al., 2008; Curran et al., 2014; analysis of RNA-SEQ data, Sessions et al., 2016) , but the earliest evidence of a mature circadian clock (maintained in DD) in a *Xenopus laevis* organ is melatonin secretion in the pineal gland during tailbud stages (1.3dpf, Green et al., 1999). Since the circadian clock coordinates physiology and gene expression with the environment in adults, we wonder whether maturation of the circadian clock in the pineal and later in the eye is particularly important for survival during early development.

We were surprised when stage 50 hearts (15 dpf) did not display rhythmic expression of additional circadian genes. In general, circadian genes are expressed at low levels in the embryo and *bmal1* has a particularly low amplitude rhythm ( Figure 1C ). For these reasons, we used a large number of hearts per time point (20 total). It is possible that pooling samples caused increased variation within each sample leading to dampening of the overall signal for *bmal1* . It is also possible, that post-transcriptional regulation of circadian genes presages rhythmic transcription of specific oscillator genes. In zebrafish embryos, *per1* transcription is rhythmic while both *clock1* and *bmal1* are not. Rhythmic *per1* transcription is abolished in the presence of dominant negative *clock1* suggesting that post-transcriptional regulation of *clock1* drives rhythmic *per1* expression in zebrafish embryos (Dekens and Whitmore, 2008) . In mice, posttranscriptional suppression of Clock protein is also linked to maturation of the circadian clock (Umemura et al., 2017) . But, we also did not detect a significant rhythm of a circadian output gene that is highly expressed in the heart ( *noct* ; Figure 1K ; Curran et al., 2008), therefore we were confident that the heart did not have a fully mature molecular circadian clock.

We were also surprised that heart rate displayed time of day dependent changes at times when the molecular circadian clock is not detectable (3.2 and 3.625 dpf) or only displays rhythmic expression of two components (5.5dpf; Figure 1 ). We hypothesize that early maturation of the circadian clock in neuronal organs may direct rhythmic changes in embryonic physiology while peripheral organ circadian clocks are maturing (Patke et al., 2020) . Although we cannot rule out that the heart itself could detect and respond directly to light similar to zebrafish (Whitmore et al., 2000; Carr et al., 2006; Weger et al., 2011) . Repeating these experiments in constant darkness could show that 24-hour changes in heart rate can be maintained in constant conditions, but would not differentiate between intrinsic regulation by the heart itself, regulation via the pineal, eye, and SCN, nor the ability to directly respond to light.

## Methods

**Obtaining embryos:** Adult *Xenopus laevis* were housed in a 12-hour light dark cycle at 19 ^o^ C (University of Wisconsin, IACUC-FY2018-2019-001). For each experiment at least 6 females were injected with Human Chorionic Gonadotropin (500units, Chorulon) in the morning, midday, and evening to support timed fertilizations during the subsequent day. Testes were dissected from males that had been anaesthetized using 0.05% Ethyl 3-aminobenzoate, methansulfonic acid salt (Acros Organics CAS 886-956-2). Testis was maintained in 1X MMR and 100ng/ml gentamycin at 4 ^o^ C. Pieces of testis were macerated and used to fertilize eggs. The embryos were de-jellied using 2% cysteine (pH8) and cultured in 1/3X MBS.

We used the following conditions to obtain embryos at three time points during the day and night, (Zeitgeber Timepoint; ZT1, 5, 9, 13,17, and 21) that were approximately the same developmental age. We fertilized embryos at six different times and cultured them in opposite light-dark cycles offset by one hour. When *Xenopus* embryos are cultured at 18 ^o^ C developmental stages 38, 39.5, and stage 40 are approximately 12 hours apart. Culture of embryos in opposite light dark cycles at 18 ^o^ C allowed us to harvest eyes at each developmental stage from each light regime to represent each developmental stage at ZT1, ZT5, ZT9, ZT13, ZT 17, and ZT21.

**Embryonic Eye Dissection:** Dissections were performed by KC to obtain eyes that were Nieuwkoop and Faber (NF) stage 38, 39.5, and 40 at each Zeitgeber Time (ZT; Niewkoop and Faber, 1994). Samples were collected in duplicate, 15 eyes from 15 individuals per sample. All dissections were completed and tissues flash frozen within one hour of removal of the embryos from the incubator. The speed of dissection was critical to make sure eyes were frozen before changes in gene expression due to a change in light conditions were detectable. Samples were flash frozen and stored at -80 ^o^ C. The process outlined in this paragraph was repeated once more with similar results at each stage.

**Embryonic Heart Dissection:** Harvest of embryonic hearts was similar in experimental design except that embryos were older when harvested at each ZT (stage 47 or stage 50). When the embryos reached the proper stage of development dissections were performed at each ZT by five undergraduate researchers and KC. Heart samples were collected in triplicate, 20 hearts per sample. Eyes were taken at each time point as a positive control (2 samples, 15 eyes per sample from 15 individuals).

**RNA isolation, Reverse transcription, and qPCR:** RNA was extracted using RNAzol (Molecular Research Center, Inc). RNA was further purified using one BAN (4-Bromoanisole, Molecular Research Center, Inc., BN 191) extraction followed by isopropanol precipitation. Genomic DNA was removed using Invitrogen Turbo DNA-free kit (AM1907). We further purified the RNA using two more BAN extractions and ethanol precipitation. These extra BAN extractions were necessary to decrease background in our qPCR. The RNA was quantified using a Nanodrop 2000, reverse transcription (RT) Applied Biosystems, 4368814). -RT control reactions were also performed.

Next, we determined the relative quantitation of target gene transcripts in each sample using qPCR (ABI Power SYBR Green Master Mix; ABI 7300). *eef1a1.L* and *rpl8.S* were used as reference genes for the eye experiments, while only *eef1a1.L* was used in the heart, as *rpl8.S* was not detectable (Ŝindelka et al., 2006). All qPCR reactions were performed in triplicate. Each primer set was validated by creating a standard curve consisting of 6-8 two-fold serial dilutions of RT reactions from a stage 40 whole embryo. qBASE was used to calculate the efficiency (1.91-2.08), slope (-3.13 to -3.34) and R ^2^ (0.99) for each curve (Pabinger, et al., 2009) . Each set of samples (ZT1,5,9,13,17,21) was diluted so that it amplified within the linear range of the respective standard curve. The primer sequences were designed using Primer3Plus (Untergasser et al. 2007) . The forward (F) and Reverse (R) Primer sequences used for qPCR are presented in Table 1. The specificity of the primer sequences was verified using BLAST analysis of the *Xenopus* genome (Xenbase; Bowes, et al. 2010). During amplification, the final annealing temperature for *noct, dbp,* and *per1* used was 60°C, while for *bmal1, nr1d1* and *aanat* it was 57°C. Dissociations were always performed to check for non-specific amplification, especially in no template controls (NTC, water) and RT- samples. Data was only included here if the RT- and water controls were negative.

**qPCR Data Analysis:** An online program, qBase (QPCR), was used for relative quantitation of each sample and primer set (Pabinger, et al., 2009) . To determine if changes in gene expression were rhythmic, we combined experimental replicates (6 time points/experiment) into a single excel document as a continuous time course (12-18 time points) and analyzed the pattern of gene expression using CircWave v1.4 software (courtesy of Dr. Roelof Hut; http://www.euclock.org). Graphs of gene expression were created using MS Excel ^TM^ .

**Heart Rate:** *Xenopus* embryos were cultured in a 12-hour light dark cycle at 19 ^o^ C. Observations of heart rate were taken using a Leica S4E stereoscope in white light (day) or red light (night) in an environmental chamber maintained at 19 ^o^ C (Percival). The timing of fertilizations and methods of culture were similar to those used for the eye and heart dissections. At each developmental time we counted the number of heart beats for 15 seconds twice and multiplied by four to determine the beats per minute for each tadpole. In these experiments, embryos were assessed for 3 consecutive days to obtain three different developmental ages (stage 40/41; stage 43/44; stage 45-47). The first experiment included 25-31 tadpoles per time point. The second experiment included 24-25 tadpoles per time point. Tadpoles were analyzed for pattern of heart rate change over time using CircWave v1.4 software (courtesy of Dr. Roelof Hut; http://www.euclock.org).

## Reagents

**Table:** 

**Table 1. qPCR Primer sequences**				
Gene Name	Symbol	NCBI Gene ID	Forward Primer (5'-3')	Reverse Primer (5'-3')
*eukaryotic translation elongation factor 1 alpha 1*	*eef1a1.L*	108704161	taccagttggtcgtgtggaa	gtaagggcttcatggtgcat
*nuclear receptor subfamily 1 group D member 1*	*nr1d1.L*	444369	tcccacctgccacccccaaa	acacggggctggtgcgattg
*basic helix-loop-helix arnt like 1*	*bmal1.S*	497632	taccttggcctttgtgatcc	tggcccctatgttttactgc
*period circadian regulator 1*	*per1.L*	373754	tgccctgtcctgcgtcaaaca	tgggaatcatcaataggccactgc
*D site of albumin promoter (albumin D-box) binding protein*	*dbp.L*	108696680	accccctccctcagaatggaaca	ggggcatgagtgatgcagaggc
*nocturnin*	*noct.L*	378568	agggatacctgtcagcgaga	aggctcagactggtcatgct
*aralkylamine N-acetyltransferase*	*aanat.S*	100335045	ctgtcggaccctgtgacat	acaggggtgaacatgggag
*ribosomal protein L8*	*rpl8.S*	380157	gatgccccagctggtcgcaa	tctttgtaccgcgcagacgacc
